# “Feature Papers in Photochemistry and Photocatalysis”

**DOI:** 10.3390/molecules30214217

**Published:** 2025-10-28

**Authors:** Barbara Bonelli

**Affiliations:** 1 Department of Applied Science and Technology and INSTM Unit of Torino-Politecnico, Corso Duca deli Abruzzi 24, I-10129 Turin, Italy; barbara.bonelli@polito.it; 2 Interdepartmental PolitoBIOMed Lab, Corso Duca deli Abruzzi 24, I-10129 Turin, Italy

We all recognize the importance of photochemistry and photocatalysis in daily life. Consider processes like photosynthesis and Vitamin D production, as well as self-cleaning coatings and paints—examples of how light benefits our lives and the environment.

In the Special Issue “Feature papers in photochemistry and photocatalysis” that I edited with Prof. Dr. Hai-Yang Liu from the School of Chemistry and Chemical Engineering of the South China University of Technology, we aim to comprehensively and engagingly disseminate some scientific findings related to the exploitation of light in photochemical or photocatalytic processes through in-depth papers. The twenty-two feature papers in this Issue consist of two reviews (one on photochemistry [1] and one on photocatalysis [2]) and 20 research papers on various photochemistry [3,4,5,6,7,8,9,10,11,12,13] and photocatalysis topics [14,15,16,17,18,19,20,21,22].

In their review, Brian Musikavanhu et al. [1] overviewed applicable techniques and approaches to improve the sensitivity and selectivity of Schiff base fluorescent chemosensors for an accurate and reliable detection of toxic and heavy metal cations in various fields, including environmental monitoring, biomedical research, and industrial safety.

The review by Zhouze Chen et al. [2] addressed the photocatalytic self-Fenton technology, which combines the advantages of photocatalysis and Fenton oxidation technology through the in situ generation of hydrogen peroxide (H_2_O_2_) and interaction with iron (Fe) ions to generate a large number of strong reactive oxygen species (ROS) to effectively degrade pollutants in the environment, focusing on the state-of-the-art, prospects and challenges of g-C_3_N_4_-based photocatalytic self-Fenton systems, and the related applications, especially in, but not limited to, environmental remediation.

Concerning the photochemistry-related research papers, Hanna Rostkowska et al. [3] elucidated the effects of UV-B (λ = 305 nm) or UV-A (λ > 360 nm) excitation on N-hydroxypyridine-2(1H)-thione molecules isolated in low-temperature normal hydrogen (n-H_2_) matrices. The molecule has multiple applications in chemistry and biochemistry, as it may serve as a source of •OH radicals that can induce damage in DNA and other biomolecules. Beata Mixta et al. [4] developed hybrid fluorescent polysaccharide/silica hollow microcapsules (YC@dye@SiO_2_) with a stable and rigid framework where yeast capsules (YCs) produced from Saccharomyces cerevisiae with encapsulated fluorescent dyes were used as catalytic template guides for developing hybrid functional organic/inorganic hollow microstructures with silica deposited on their surface. Such biocompatible and biodegradable fluorescent microstructures can act as a matrix for porous fluorescent OLED biomembranes with long-term stability, as fillers, or as coating pigments. Their biocompatibility and safety make them promising candidates for use as carriers in drug delivery, imaging, and targeted therapies. Nitrilimines (R′–CNN–R″) are highly versatile intermediates in organic synthesis, particularly in regio- and stereoselective 1,3-cycloaddition reactions. They are used in general synthetic chemistry, bioorthogonal chemistry, and materials science: A. J. Lopez Jesus et al. [5] presented an experimental and theoretical study of the effects of the NH_2_ substitution at the meta and para positions on the bond-shift isomeric properties of C-phenyl-nitrilimine. Their findings offer valuable insights for future research and potential applications involving substituted nitrilimines, particularly in the design of compounds with specific electronic and structural properties. Dimethylsulfoxide is widely used in medicine as a biocompatible solvent for biologically relevant molecules and in synthetic chemistry as a low-toxicity polar solvent. Some peculiar biarylsulfoxides scaffolds exhibit photo-reactivity: Valentin Magné et al. [6] prepared and characterized three new pentafluorophenyl copper(I)–biarylsulfoxide complexes, [Cu(C_6_F_5_)]_4_L_2_, and studied their photochemical behavior, revealing some unprecedented results and the possibility of synthesizing Cu_2_O. Christopher Abelt et al. [7] conducted an experimental and computational study on Prodan derivatives, a class of amino-carbonyl disubstituted naphthalenes that are often highly fluorescent and may exhibit solvatochromic properties. Electrofluorochromic (EFC) materials, along with electrochromic (EC) materials, may have diverse applications in smart windows, optoelectronics, optical displays, etc.: Kang Le Osmund Chin et al. [8] developed an elegant approach based on a simple cross-coupling method to prepare optically transparent, heat-induced, cross-linkable styryl-functionalized EFC systems with tunable fluorescence colors and high optical transparency. Perylenetetracarboxylic diimide (PTCDI) is an n-type organic semiconductor molecule with numerous applications, including photocatalysis and field-effect transistors. Polarizability and dipole moment are crucial parameters that control the response of molecules to external electric and optical fields, as well as their physical properties and reactivity. Bulu Rahman et al. [9] explored the effects of external electric fields on the absorption and fluorescence spectra to obtain the polarizability and dipole moment of a PTCDI substituted with an octyl group (PTCDI-C8) dispersed in a polymethyl methacrylate matrix. Radosław Banasz et al. [10] developed and characterized two star-shaped viologens containing 1,3,5-substituted phenyl (1) and triphenylamine (2) central cores, and n-hexyl chains exhibiting promising optoelectronic properties as measured by spectroscopic, electrochemical, spectroelectrochemical, and luminescence methods. Both star-shaped viologens exhibit promising optical properties, and these molecules could be utilized as active materials in EC applications. Haijie Ben et al. [11] developed two novel S-heterocyclic annulated perylene diimide derivatives (PDIs), a kind of n-type semiconductor. Such molecules are crucial for the advancement of organic electronics, but the lack of knowledge about the relationship between the structure and the electrical properties of such molecules hampers the progress of PDI-based organic electronics. Their findings on the importance of the structure–function relationship in PDIs provide useful roadmaps for designing high-performance organic electronics for practical applications. MXenes are reported in the fields of energy storage, sensors, light-emitting diodes, electromagnetic shielding, and environmental applications: Ju Hee Gu et al. [12] reported the effects of the addition of redox mediators comprising I^−^, Co^3+^, and Ti_3_C_2_Tx MXene to a liquid electrolyte on the photovoltaic performance of DSSCs (Dye-Sensitized Solar Cells). The presence of Ti_3_C_2_Tx MXene in the redox mediator increased the hole collection, dye regeneration, and electron injection efficiencies. By applying time-resolved spectroscopies and time-dependent DFT calculations, Runhui Liang et al. [13] unraveled the photo-deactivation mechanisms of 3′,5′-dimethoxybenzoin (DMB) fluoride in different solvents. DMB is an essential member of the photoremovable protecting group (PRPG) and has received significant research for its efficient photo-releasing functional groups, such as nucleotides, carboxylates, and alcohols. They also provided critical insights regarding the biomedical application of DMB-based PRPG compounds.

Concerning the photocatalysis-related research papers, using an environmentally friendly approach, Kunyang Li et al. [14] designed tungsten-doped TiO_2_ with a tunable W^5+^/W^6+^ ratio via the sol–gel method. After a thorough physicochemical characterization, highlighting the effect of the synthesis conditions (i.e., type of solvent) on the photocatalysts’ properties, they successfully tested them in the photocatalytic degradation of two antibiotics, namely tetracycline and ciprofloxacin, under illumination from a xenon lamp emitting in the 170–700 nm range. Xiangxiu Lv et al. [15] developed a system based on resorcinol–formaldehyde (RF) resin and red mud (RM) that generates hydrogen peroxide (H_2_O_2_) in situ and transforms it into hydroxy radicals (•OH) for the efficient degradation of the antibiotic tetracycline under visible light irradiation, proving a possible new idea for environmental recovery in a waste-based heterogeneous photocatalytic self-Fenton system. Srijith et al. [16] produced copper selenide (β-Cu_2−x_Se) by Chemical Vapor Deposition and tested it on the photocatalytic degradation of tetracycline hydrochloride (TC-HCl). β-Cu_2−x_Se exhibited a 98% degradation efficiency, due to its layered structure, which allows for the occurrence of exposed reactive sites and is responsible for its stability, interfacial charge transfer efficiency, and visible light absorption capacity. Xiao Wang et al. [17] produced Fe-doped TiO_2_ nanofibers and tested them for the photocatalytic Fenton-like decomposition of the antibiotic tylosin (TYL) under LED illumination. Compared with the undoped TiO_2_ nanofibers (TNs), the Fe-TNs exhibited improved visible-light-driven photocatalytic Fenton-like activity with a TYL degradation efficiency of 98.5% within four hours. The enhanced TYL degradation was attributed to both a better utilization of visible light and an improved separation and migration of photogenerated electrons and holes following the introduction of Fe. Xiangyuan Kong et al. [18] designed a 2D/2D S-scheme crystalline carbon nitride (CCN)/BiOIO_3_ (BOI) van der Waals heterojunction for the efficient degradation of tetracycline under visible light irradiation and studied the degradation mechanism. The heterojunction can both accelerate the separation and transfer of interface carriers and provide sufficient active sites. The S-scheme heterojunction enhances the redox capacity of CCN/BiOIO_3_, thus providing a driving force for the degradation reaction. Zhong Xu et al. [19] synthesized silver–manganese oxide nanoparticles (Ag-Mn-NPs) through a wet chemical precipitation method and tested their photocatalytic activity for the degradation of malachite green dye under sunlight irradiation in an aqueous medium. Malachite green is a food coloring agent, food additive, medical disinfectant, and anthelmintic agent. Additionally, it finds application in industrial dyeing processes of silk, wool, jute, leather, cotton, paper, and acrylic. It is also extensively utilized as a biocide. Following the physicochemical characterization, the synergy between manganese and silver yields a photocatalyst with enhanced efficiency. Špela Slapniˇcar et al. [20] produced nanoflower-shaped TiO_2_-supported Au photocatalysts and thoroughly investigated their physicochemical properties through a multi-technique approach. They tested them for the H_2_-assisted photocatalytic reduction of NO_2_ to N_2_ at room temperature under visible-light illumination, demonstrating the prominent role of the synthesis conditions in producing effective photocatalysts. Isabel Guerrero et al. [21] deeply explored the effect of non-covalent interactions in novel cooperative photoredox systems for efficient photocatalytic oxidation of aromatics and alkenes in water to produce epoxides, a class of intermediate and basic components used to obtain more elaborate chemical products in both organic synthesis and in the industrial production of fine and bulk chemical products. Maurizio Ballico et al. [22] prepared neutral cyclometalated [Ru(η_2_-OAc)(NC-tpy)(PP)] and cationic [Ru(η_1_-OAc)(NNN-tpy)(PP)]OAc (PP = diphosphine) terpyridine complexes for photocatalytic applications in asymmetric transfer hydrogenation of acetophenone. Interestingly, the same complexes exhibit cytotoxic activity toward anaplastic thyroid cancer cell lines.

Examining the papers published in this issue collectively, it becomes clear that these works consistently employ an interdisciplinary approach and advanced experimental and computational methodologies to study novel molecules, materials, and processes that could have a significant impact on medicine, sensing, optoelectronics, environmental remediation, materials science, the chemical industry, displays fabrication, photovoltaics, and related fields. The success of this Issue and the high level of the published papers encouraged us to propose a Second Edition, which is now open for submission at this link https://www.mdpi.com/journal/molecules/special_issues/995XTCAYOO “URL (accessed on the 14 July 2025)”.
